# Short-term effects of COVID-19 vaccines on cardiac biomarkers: A comparative study between Pfizer/BioNTech and Sinopharm

**DOI:** 10.5339/qmj.2024.73

**Published:** 2024-12-30

**Authors:** Yousaf Dawood, Saddam M. Abed, Yousif H. Khalaf

**Affiliations:** ^1^Department of Pharmacology and Toxicology, College of Pharmacy, University of Anbar, Ramadi, Iraq; ^2^Al-Ramadi Teaching Hospital for Women and Children, Anbar Health Directorate, Ramadi, Iraq; ^3^Department of Clinical Laboratory Sciences, College of Pharmacy, University of Anbar, Ramadi, Iraq *Correspondence: Yousif H. Khalaf. Email: ph.yhks1980@uoanbar.edu.iq

**Keywords:** COVID-19 vaccines, cardiac biomarkers, neutralizing antibodies, side effects

## Abstract

**Objectives:** The aim of this study was to investigate the potential cardiac side effects associated with Pfizer/BioNTech and Sinopharm vaccines.

**Methods:** A total of 200 healthy volunteers were enrolled after receiving two doses of Pfizer/BioNTech or Sinopharm vaccine 21 days apart. In addition, 100 healthy, unvaccinated individuals were included as a control group. Cardiac biomarkers such as cardiac troponin-I (cTnI), creatinine kinase–myocardial band (CK-MB), and myoglobin (MYO) were measured 4 days after receiving the second dose of vaccine. In addition, the SARS-CoV-2 neutralizing antibody levels of all participants were also determined.

**Results:** Post-vaccination findings in both vaccinated groups were comparable. There was no statistically significant difference in cTnI, MYO, and CK-MB levels between the Pfizer/BioNTech and Sinopharm vaccine groups. Furthermore, our results showed that the levels of SARS-CoV-2 neutralizing antibodies stimulated by the Pfizer–BioNTech vaccine were significantly higher (171.19, *p* < 0.001) compared to the Sinopharm vaccine (70.14). Owing to the successful efficacy of the vaccine and based on the available data, COVID-19 vaccines remain the best option to eliminate the pandemic and its complications.

**Conclusion:** Our study shows that both Pfizer–BioNTech and Sinopharm vaccines are safe for the heart in the short term, with no significant changes in cardiac biomarkers observed four days after vaccination. The findings suggest that these vaccines are effective and do not pose an immediate risk to cardiovascular health within the observed time frame. However, further research is required to assess the long-term cardiac safety profiles of these vaccines.

## INTRODUCTION

The coronavirus disease (COVID-19) outbreak caused by the SARS-CoV-2 virus is classified as a pandemic that affects different regions of the globe.^1^ The outbreak of the pandemic led to far-reaching psychological, health, and economic challenges. Approximately one year after the onset of the COVID-19 crisis, the World Health Organization issued the first EUL (Emergency Use Listing) for a vaccine.^2^ The first approval for a COVID-19 vaccine was granted to the American company Pfizer in collaboration with the German biotechnology company BioNTech (Pfizer–BioNTech), with the FDA endorsing its emergency application on December 11, 2020. Subsequently, the Moderna vaccine received a similar approval on December 18, 2020. These vaccines represented the initial offerings on the market that were both efficacious and safe.^2,3^ Then, it was followed by the discovery of numerous vaccines worldwide.

A virus particle consists of a single-stranded positive-sense RNA enclosed by an envelope containing envelope proteins, membrane proteins, and spike proteins. The virus enters the lung cells via the S1 and S2 subunits. S1 mediates linking to the angiotensin-converting enzyme 2 (ACE2) receptors of the host cells, while S2 mediates cell fusion.^4^ Humans mainly obtain neutralizing antibodies via viral contagion, plasma treatment, and vaccination. The detection of SARS-CoV-2 neutralizing antibodies is critical for assessing both the patient's health status and vaccination outcomes. The neutralizing antibody interacts with the receptor-binding domain of the S protein to mainly prevent the virus from binding to ACE2.^5^ According to a recent study, various specific neutralizing antibodies can be separated from restored blood samples of SARS-CoV-2 infected people.^6^

Many COVID-19 vaccines were created to protect against the SARS-CoV-2 virus. The main goal of these vaccines is to reduce the severity of the infection and prevent secondary infections. Through various mechanisms, COVID-19 vaccines promote both innate and adaptive immunity, thereby providing protection against SARS-CoV-2 infection.^7^ Serological diagnostic assays are used to evaluate the efficacy of the COVID-19 vaccine. Assays to determine the immune response to COVID-19 are based on the identification of antibodies against SARS-CoV-2 nuclear antigens and spike antigens. These tests are also functional assays to measure in vitro infectivity.^8^ The majority of COVID-19 vaccines aim to stimulate the production of antibodies against the spike protein of the virus.^2^

Clinically, the combined detection of cardiac troponin-I (cTnI), cardiomyocyte creatinine kinase–myocardial band (CK-MB), and myoglobin (MYO) could improve diagnostic specificity and sensitivity.^9^ According to recent studies, cardiac complications associated with COVID-19 vaccines include pericarditis, myocarditis, and myocardial infarction.^10–12^ Fazlollahi et al. reported that young males with a median age of 21 years had the highest incidence of myocarditis and significant increases in CK-MB, cTnI, and NT-proBNP levels following the administration of mRNA COVID-19 vaccines.^13^ A recent study showed a reverse takotsubo cardiomyopathy as a cause of acute chest pain in a young woman following COVID-19 vaccination, resulting in elevation of serum cTnI and CK-MB levels.^14^ Furthermore, in a large Israeli healthcare system, the incidence of myocarditis within 42 days of receiving the first dose of the BNT162b2 mRNA vaccine was estimated to be 2.13 cases per 100,000 people. The highest rate was observed in males aged 16–29 years. Most myocarditis cases were classified as mild to moderate.^15^ In the same context, the Israeli Ministry of Health documented 148 cases of myocarditis between December 2020 and May 2021, mostly affecting males aged 16–30 years, after receiving the second dose of the mRNA COVID-19 vaccine. This report suggested a potential association between the vaccine and myocarditis in this population group.^16^ The incidence of myocarditis seemed to increase approximately 3–5 days after receiving the second dose of the mRNA vaccine. This observation is consistent with several studies that have documented an increased risk during this period.^15,17^ In contrast, there are currently no studies on cardiac complications of inactivated SARS-CoV-2 vaccination.^18^

Relatively rare severe adverse reactions to vaccines may not be detected in COVID-19 clinical trials due to the small random sample, limited inclusion criteria, short duration of follow-up, and study participants involved, who may differ from the individuals eventually receiving the vaccines.^19^ Recently, several studies have examined the potential impact of some COVID-19 vaccines on body functions, including mRNA vaccines such as Pfizer/BioNTech and Moderna, as well as inactivated vaccines such as Sinopharm, Covaxin, and others such as Novavax, Sputnik, and AstraZeneca.^20,21^ Two vaccines were used in this study: the traditional inactivated vaccines such as Sinopharm and the mRNA vaccines from Pfizer–BioNTech. The aim of the present study was to investigate the potential side effects of these vaccines on cardiac biomarkers such as cTnI, CK-MB, and MYO. It also addressed the measurement of SARS-CoV-2 neutralizing antibody levels after vaccination against COVID-19 in order to detect anti-SARS-CoV-2 antibodies produced against this virus.

## METHODS

### Study design

The study was randomized and included 200 male and female healthy Iraqi adults who received two doses of the Pfizer–BioNTech vaccine or the Sinopharm COVID-19 vaccine. The period between the two vaccinations was 21 days for all included participants. The age range was between 20 and 65 years. The study used a simple randomization method to allocate participants to the Pfizer–BioNTech and Sinopharm vaccine groups. A computer-generated random number sequence was used to ensure unbiased assignment. The participants were randomly divided into three groups: 100 individuals (with a mean age of 33.4 ± 5.30 years) received the Pfizer–BioNTech vaccine, another 100 (with a mean age of 34.8 ± 4.56 years) received the Sinopharm vaccine, and the remaining 100 individuals (with a mean age of 32.1 ± 6.11 years) were assigned to a control group.

The duration of this study was between January and May 2022. The study was conducted after the voluntary vaccination of participants in the hospitals of the Iraqi Ministry of Health. This study followed the criteria of the Declaration of Helsinki and was approved by the Research Ethical Committee of the University of Anbar (approval no. 71/ 27-06-2022). Before participating in this study, each enrolled participant provided written informed consent. A standardized questionnaire was used to collect demographic information, including age, gender, residential location, medical history, vaccine type, and any associated side effects.

### Inclusion and exclusion criteria

The study participants were male and female healthy Iraqi adults who were enrolled and vaccinated with either the Pfizer–BioNTech or Sinopharm vaccine during the study. Cardiac biomarkers were measured after participants received their second dose of vaccine and signed an informed consent form. The study excluded people with previously diagnosed cardiac conditions, those who were immunocompromised, and those taking immunosuppressive medications. In addition, individuals who were previously infected with COVID-19, those who had only received the initial injection of the COVID-19 vaccines mentioned above, and those who did not receive the two doses within the recommended time frame were excluded.

### Sampling collection

The sample was collected four days after the second vaccination because most cases of myocarditis occur within 3–5 days after vaccination, particularly after the administration of the second dose, as previously reported by Witberg et al. and Shay et al.^15,17^ Five milliliters of whole blood samples were collected from the studied groups and the control group. The samples were centrifuged at 300 × g for 10 min. Serum samples were then collected and stored at – 20°C until used for laboratory analysis.

### Measurement of cardiac biomarkers and SARS-CoV-2 neutralization antibodies

The NIPIGON HEALTH CORP (Automated Electrochemiluminescence Immunoassay Analyzer, Ontario, Canada, Model: Robot R1) was used to measure cardiac biomarkers (cTnI, CK-MB, and MYO) and SARS-CoV-2 neutralization antibodies. The duration of an assay was 11 min for cTnI and 9 min for CK-MB, MYO, and SARS-CoV-2 neutralization antibodies. cTnI concentrations were determined using two cTnI-specific monoclonal antibodies that target two different cTnI epitopes.^22^ CK-MB concentrations were determined using CK-MB-specific monoclonal antibodies.^23^ MYO concentrations were measured using two MYO-specific monoclonal antibodies.^24^ SARS-CoV-2 neutralizing antibodies have been found to be naturally produced in vaccinated individuals.^25^ A calibration was performed on the NIPIGON ROBORT1. The titration solution was supplemented with 300 μL of serum. Fluorescence was quantified at 350 nm after the reaction had run for 20 min. The amount of antibodies present in the sample under investigation is directly related to the intensity of this fluorescence. As a result, test values were recorded and the outcomes were automatically calculated based on the values stored in the memory of the devices.

### Calculation of sample size

A power analysis was performed to determine the appropriate sample size for this study. The sample size was calculated to ensure adequate power to identify a statistically significant effect, with an alpha (α) value of 0.05. The α value of 0.05 was selected because it is a widely accepted threshold for statistical significance (95% confidence level), balancing the risk of false positives and false negatives. By setting the α value at 0.05, we aim to maintain a 5% probability of falsely rejecting the null hypothesis when it is true, which is a standard practice in many scientific fields.

### Statistical analysis

Data are presented as mean ± SE and indicative of three different investigations. GraphPad Prism 5.01 software was used to analyze the data. A two-tailed, unpaired t-test was used to compare differences in SARS-CoV-2 neutralizing antibodies between the Pfizer/BioNTech and Sinopharm vaccine groups. Significant *p* values were defined as values below 0.05.

## RESULTS

### Evaluation of cardiac biomarkers in Pfizer and Sinopharm vaccine recipients

**Figure 1.**
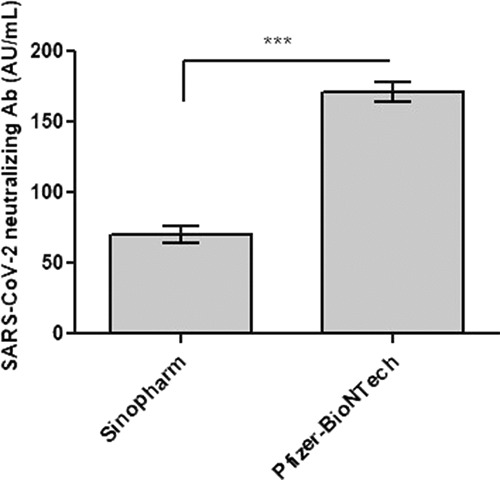
SARS-CoV-2 neutralizing antibodies in 100 fully vaccinated recipients with Pfizer–BioNTech vaccine versus 100 fully vaccinated recipients with Sinopharm vaccine. Each value is the mean ± SE of three experiments conducted independently. A t-test was used to evaluate the results. ***p < 0.001: SARS-CoV-2 neutralizing antibody levels were significantly higher in the Pfizer–BioNTech-vaccinated group than in the Sinopharm-vaccinated group.

Cardiac biomarkers were assessed in 300 participants; 100 subjects received the Pfizer–BioNTech vaccine, 100 subjects received the Sinopharm vaccine, and a negative control group of 100 subjects were unvaccinated. Table 1 shows that participants who received the Pfizer–BioNTech or Sinopharm vaccine maintained normal cTnI, CK-MB, and MYO levels. Figure 1 shows no change in cTnI expression levels after vaccination with Pfizer–BioNTech or Sinopharm compared to the unvaccinated group. Furthermore, CK-MB and MYO activity was found to increase in individuals vaccinated with Pfizer–BioNTech and Sinopharm. However, this increase was not statistically significant, as shown in Figure 1. The data showed that the Pfizer–BioNTech and Sinopharm vaccines had no effect on cardiac biomarkers in the studied groups. Fever, headache, fatigue, joint pain, muscle pain, dyspnea, cough, nausea, dizziness, appetite impaired, rhinorrhea, and redness and swelling at injection sites were the most common adverse events with the use of the COVID-19 vaccines. In contrast, there were no complications such as myocarditis and pericarditis, stress cardiomyopathy, acute myocardial infarction, and arrhythmia.

### Identification of SARS-CoV-2 neutralizing antibodies following the Pfizer–BioNTech or Sinopharm vaccine

Further investigations measured the levels of SARS-CoV-2 neutralizing antibodies in individuals vaccinated with Pfizer–BioNTech and Sinopharm vaccines to determine the efficacy of these vaccines against COVID-19 compared to the control group. As expected, the control group without vaccination did not induce SARS-CoV-2 neutralizing antibodies. In contrast, the data showed that the Pfizer–BioNTech and Sinopharm vaccines effectively induced SARS-CoV-2 neutralizing antibodies at 171.19 AU/mL and 70.14 AU/mL, respectively (Table 1). Furthermore, Figure 1 shows that the Pfizer–BioNTech vaccine significantly induced the levels of SARS-CoV-2 neutralizing antibodies compared to the Sinopharm vaccine (*p* < 0.001). This result indicates that individuals vaccinated with Pfizer–BioNTech produced a stronger immune response to SARS-CoV-2 than individuals vaccinated with Sinopharm.

## DISCUSSION

Diagnosis and management of heart diseases pose a challenge in the clinic. Clinical heart disease often occurs as a result of a viral infection or vaccination.^26^ SARS-CoV-2 vaccines demonstrated remarkable safety and efficiency in the population. This triggered a robust immune response, limiting the viral infection and its consequences.^27^ However, some of the potential adverse effects of these vaccines should not be overlooked. The most common cardiac issues following COVID-19 vaccination were myocarditis and pericarditis, with some cases of stress cardiomyopathy, acute myocardial infarction, and arrhythmia.^18,28^

According to recently published findings, myocarditis occurs infrequently, with a case rate of less than 106 per million COVID-19 vaccine doses. Cardiac troponin levels were significantly higher in almost all of them.^29^ This study examined cTnI, CK-MB, and MYO levels in people fully vaccinated with Pfizer–BioNTech and Sinopharm to evaluate the incidence of potential cardiac complications from these vaccines compared to unvaccinated people.

Table 1 shows that cTnI, CK-MB, and MYO levels after the total dose of the Pfizer–BioNTech and Sinopharm vaccine were within the normal range in all vaccinated and unvaccinated participants, suggesting that in the present study the vaccination used may not affect cardiac functions. Alizadeh et al. demonstrated that MYO levels were normal, while high-sensitivity troponin levels were increased in a case of myocarditis after COVID-19 vaccination.^30^ A recent study showed elevated cTnI levels (1,035 ng/L) were suggestive of myocarditis in a 20-year-old male after receiving the second injection of the Pfizer–BioNTech COVID-19 vaccine. Nevertheless, myocarditis cases following SARS-CoV-2 mRNA vaccination in young people are rare, mild, and often occur after the second vaccination injection.^31^ Another study also reported high cTnI (22 ng/mL) in a 30-year-old healthy man after two doses of the mRNA COVID-19 vaccine.^32^ In the same context, a study by Acharya et al. revealed one of the rare cases of elevated cTnI levels as a result of immune or inflammatory responses to the Pfizer–BNT162b2 COVID-19 vaccine that led to myocarditis.^33^

One study found high blood levels of CK-MB (18 IU/L) and cTnI (1008.7 pg/mL) as cardiac biomarkers in a healthy 30-year-old woman after receiving the second vaccination of mRNA COVID-19 vaccine.^14^ Furthermore, cardiac troponin-T and CK-MB levels were slightly increased in a cohort of adolescents following mRNA SARS-CoV-2 vaccination.^34^ A previous study showed that cardiac troponins are more sensitive biomarkers for identifying myocardial injury than others, such as MYO and CK-MB.^35^ On the contrary, as in our results, cardiac manifestations of the Sinopharm COVID-19 vaccine were not reported in any study.^18^

To prove the efficacy of the vaccines used in the study, the levels of neutralizing antibodies were also determined. Table 1 shows that people who received the Pfizer–BioNTech vaccine had a more robust immune response to the SARS-CoV-2 virus than people who received the Sinopharm vaccine. The Pfizer–BioNTech vaccine induced an approximately two-and-a-half-fold increase in the levels of SARS-CoV-2 neutralizing antibodies compared to the Sinopharm vaccine (*p* < 0.001, Fig. 1). A previous research showed that the efficacy of COVID-19 vaccines against the disease decreases over time.^36^ Regarding the correlation between age and neutralizing antibody levels, a recent study found that levels of neutralizing antibodies decrease with age, but there was no statistically significant difference.^25^. Neutralizing antibody levels in vaccinated subjects also predicted a lower risk of intensive care unit admission, serious illness, and fatality.^37^

### Strengths and limitations

The strengths of this study include a comparative analysis of the Pfizer–BioNTech and Sinopharm vaccines, highlighting differences in efficacy and side effects, which is valuable for public health decisions. Additionally, the diverse population enables a comprehensive analysis of vaccine efficacy across different age groups, genders, and health conditions, providing specific insights into vaccine performance in the local context, considering factors such as prevalent variants, healthcare infrastructure, and population health behaviors.

However, the study has several limitations. Firstly, the small sample size may limit the generalizability of the results. A larger, more representative sample is essential for drawing robust conclusions. Additionally, the emergence of new COVID-19 variants could affect the efficacy of the vaccine, for example through variant-specific immune responses that may elicit different immune responses compared to the original strains targeted by the vaccines. This could affect the levels and types of cardiac biomarkers observed after vaccination. Furthermore, the efficacy of both Pfizer/BioNTech and Sinopharm vaccines against new variants may vary, potentially influencing the comparative results of cardiac biomarkers. Factors such as pre-existing health conditions, socio-economic status, and access to healthcare can also influence outcomes and may not be fully accounted for. Finally, a short follow-up period may not capture long-term efficacy and side effects, which are crucial for a comprehensive understanding of the vaccine's impact.

By evaluating the strengths and limitations, this study provides crucial insights into the effectiveness of Pfizer and Sinopharm vaccines in Iraq, contributing to the development of more informed public health strategies.

## CONCLUSIONS

In conclusion, normal levels of cardiac biomarkers in fully vaccinated study participants demonstrated that cardiac issues associated with mRNA COVID-19 vaccines are rare. Furthermore, Pfizer–BioNTech outperformed the Sinopharm vaccine in terms of vaccine efficiency. The study provides evidence that both vaccines had no effect on the cardiac enzymes tested.

### Competing interests

The authors have no conflicts of interest to declare.

### Ethical approval

Ethical approval was obtained from the Research Ethical Committee of the University of Anbar under ID 71 on June 27, 2022.

### Informed consent

Written consent was obtained from volunteers for publication of this article.

### Authors’ contribution

**Yousaf Dawood:** Conceptualization, Methodology, Data curation, Writing – Original draft, Formal analysis, Funding acquisition, Investigation, Resources, Software, Validation, and Visualization. **Saddam M. Abed:** Conceptualization, Methodology, Data curation, Formal analysis, Funding acquisition, Investigation, Resources, Software, Validation, and Visualization. **Yousif H. Khalaf:** Conceptualization, Methodology, Data curation, Supervision, Writing – Original draft, Writing–review and editing, Formal analysis, Funding acquisition, Investigation, Project administration, Resources, Software, Validation, and Visualization. All authors read and approved the final version of the manuscript.

## Figures and Tables

**Table 1.** Cardiac biomarkers and SARS-CoV-2 neutralizing antibodies of recipients who received two doses of Pfizer–BioNTech or Sinopharm vaccines versus those who were unvaccinated (mean ± SE, *n* = 100).

Parameter	Control		Pfizer-BioNTech		Sinopharm	

	Mean	SE	Mean	SE	Mean	SE

cTnI (ng/ml)	0.01	0.00	0.01	0.00	0.01	0.00

CK-MB (ng/ml)	1.93	0.18	1.87	0.18	2.39	0.20

MYO (ng/ml)	40.00	1.19	44.97	4.45	43.50	6.59

SARS-CoV-2 neutralizing antibodies (AU/ml)	0.00	0.00	171.19	12.01	70.14	10.43

